# Concurrent therapy with immune checkpoint inhibitors and TNFα blockade in patients with gastrointestinal immune-related adverse events

**DOI:** 10.1186/s40425-019-0711-0

**Published:** 2019-08-22

**Authors:** Yousef R. Badran, Justine V. Cohen, Priscilla K. Brastianos, Aparna R. Parikh, Theodore S. Hong, Michael Dougan

**Affiliations:** 1 Department of Medicine, Massachusetts General Hospital, Boston, MA 02114 USA; 2 Harvard Medical School, Boston, MA 02115 USA; 3 Division of Oncology, Department of Medicine, Massachusetts General Hospital, Boston, MA 02114 USA; 4 Department of Radiation Oncology, Department of Medicine, Massachusetts General Hospital, Boston, MA 02114 USA; 5 Division of Gastroenterology, Department of Medicine, Massachusetts General Hospital, Boston, MA 02114 USA

**Keywords:** Checkpoint inhibitor, PD-1, CTLA-4, TNFα, Infliximab, Immune related enterocolitis, Immune related adverse events

## Abstract

**Background:**

Immune checkpoint inhibitors (ICI) have demonstrated remarkable efficacy as cancer therapeutics, however, their use remains limited due to the development of immune related adverse events (irAEs). Immune related enterocolitis (irEC) is among the most common severe irAEs leading to the discontinuation of ICIs. Inhibitors of tumor necrosis factor alpha (anti-TNFα) have been used to treat irEC. Recent animal studies have shown that concurrent treatment with anti-TNFα and ICIs improves tumor responses and decreases colitis severity. This approach has not yet been studied in prospective trials in humans. Here we describe, for the first time, the outcomes of patients who were treated concurrently with anti-TNFα and one or two ICIs.

**Case presentations:**

Five patients with different primary malignancies were treated with ipilimumab/nivolumab (2 patients), pembrolizumab (1 patient), ipilimumab (1 patient), or cemiplimab (1 patient). All patients developed irEC within 40 days of their first ICI dose. The patients presented with a combination of upper and lower gastrointestinal symptoms and subsequently underwent upper endoscopy and/or lower endoscopy. Endoscopy results demonstrated a spectrum of acute inflammatory changes across the gastrointestinal tract. Steroid therapy was used as first line treatment. To prevent prolonged steroid use and recurrence of gastrointestinal inflammation after resumption of cancer therapy, patients were treated concurrently with infliximab and ICI. Patients tolerated further ICI therapy with no recurrence of symptoms. Repeat endoscopies showed resolution of acute inflammation and restaging imaging showed no cancer progression.

**Conclusions:**

Concurrent treatment with anti-TNFα and ICI appears to be safe, facilitates steroid tapering, and prevents irEC. Prospective clinical trials are needed to assess the outcomes of this treatment modality.

## Background

Monoclonal antibodies targeting the immune regulatory “checkpoint” receptors programmed death receptor 1 (PD-1), its ligand, PD-L1, and cytotoxic T cell associated antigen 4 (CTLA-4) have demonstrated remarkable efficacy against advanced cancers [1]. The natural roles of these immune checkpoints include preventing T cell over-activation, inducing anergy, maintaining peripheral immune tolerance, and contributing to T cell exhaustion in local inflammatory environments [2, 3]. By inhibiting these regulatory receptors, immune checkpoint inhibitors (ICIs) boost the anti-tumor effector functions of T cells [2]. Simultaneously, ICI-induced loss of tolerance leads to off-target immune related adverse events (irAEs) [4]. The frequency and severity of these events are an important limitation of immunotherapy, leading to treatment interruption and even discontinuation [5].

Immune related (entero) colitis (irEC) is among the most common severe irAEs leading to the discontinuation of immunotherapy [6–9]. High dose glucocorticoids are the first line management of immune mediated enterocolitis [10–13]. Current guidelines recommend continuation for at least 4 to 6 weeks after resolution of irEC [10–13]. Not infrequently, patients are intolerant to steroid tapers or require prolonged courses of glucocorticoids for symptom control. Prolonged use of glucocorticoids is associated with multiple complications that include serious infections, hyperglycemia, osteoporosis, and altered mental status. In patients with irEC who fail steroid tapers or are steroid unresponsive, inhibitors of tumor necrosis factor alpha (anti-TNFα) have been used to suppress mucosal inflammation [14, 15]. Retrospective studies of patients who received anti-TNFα agents for irEC have shown that compared to steroids, these agents lead to more rapid symptomatic improvement and a shorter duration of steroids without affecting time to ICI treatment failure or overall survival [6, 14–16]. For patients who require anti-TNFα agents to control irEC, ICIs are generally discontinued out of concern for recurrent, potentially treatment refractory, colitis. Recent work in mouse models of cancer has shown that co-administration of ICI and anti-TNFα upfront led to improved tumor responses and decreased colitis severity, an approach that has not yet be studied in prospective trials in humans [17, 18]. In this report, we present our institutional experience on patients with different malignancies who were treated concurrently with anti-TNFα and single or combination ICIs.

## Patients and methods

Included in this series are patients evaluated and treated at the Massachusetts General Hospital who were referred to the gastroenterology service for new gastrointestinal complaints (abdominal pain, protracted nausea and vomiting, or diarrhea) that arose during treatment with one or more ICIs. ICIs were administered as standard-of-care or as a part of a clinical trial. Details of the patients’ medical history, malignancy, and prior cancer therapeutics were reviewed in the chart. Data pertaining to ICI use and irEC development and management include doses of ICI, presenting grades of diarrhea and colitis, glucocorticoid dose and number of steroid taper attempts, infliximab dose and frequency, doses of infliximab to clinical remission, and doses of ICI concurrently administered with infliximab. Diarrhea and colitis were graded using the Common Terminology Criteria for Adverse Events (CTCAE) version 5.0 at disease onset [19]. Upper endoscopy and/or colonoscopy/flexible sigmoidoscopy were performed as clinically indicated at the Massachusetts General Hospital Endoscopy Unit. Consent was obtained from all patients. Pathology was reviewed by board certified pathologists. Radiologic imaging data were obtained as indicated by the treatment protocol. This retrospective study was approved by the institutional review board of the Massachusetts General Hospital.

## Results

### Patient 1

**Fig. 1:**
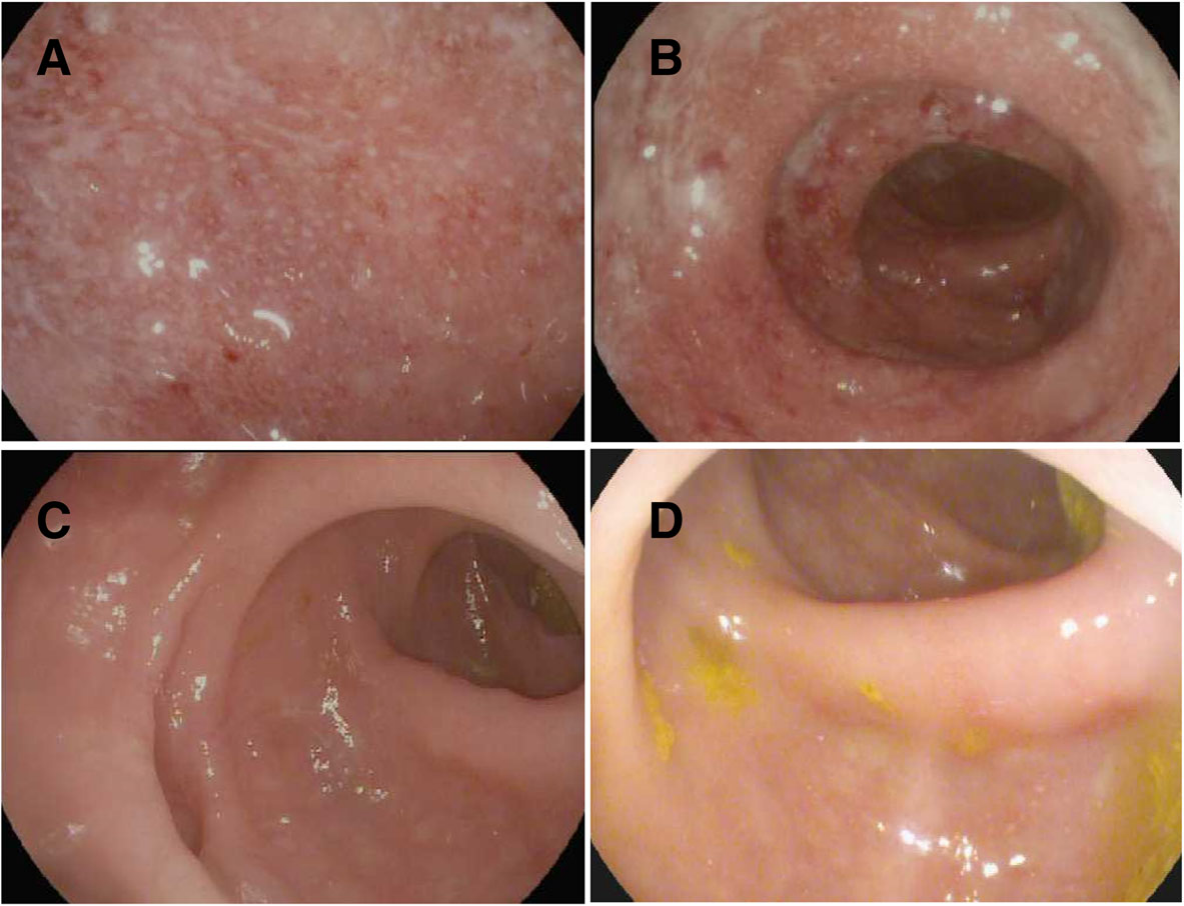
Infliximab and pembrolizumab for segmental colitis in meningioma. **a** – **d** (Images taken from the sigmoid colon during endoscopic evaluation. **a** Diagnosis of recurrent SCAD. **b** After completion of antibiotics and on prednisone. **c** Infliximab and prednisone co-treatment. **d** Infliximab and pembrolizumab co-treatment

Patient 1 is a 70-year-old man with a past medical history of segmental colitis associated with diverticulosis (SCAD) who was diagnosed at the age of 73 with a right vestibular schwannoma and a large bifrontal atypical meningioma. At diagnosis, he was treated with subtotal resection and postoperative proton therapy. Two years later, he presented with recurrent bitemporal extra-cranial soft tissue meningioma treated with resection and radiation therapy. He was then started on pembrolizumab (PD-1 inhibitor) monotherapy and received two doses (Table 1). After receiving his second dose of pembrolizumab, he developed intermittent rectal bleeding without urgency, diarrhea, abdominal pain, cramping or bloating. He underwent a colonoscopy that showed endoscopic and histologic features of active colitis (Fig. 1). Based on these findings, he was treated with prednisone 60 mg, azithromycin, and metronidazole for a 7-day course that led to symptomatic improvement. With tapering of the steroids, his rectal bleeding recurred which prompted a repeat flexible sigmoidoscopy that demonstrated persistent inflammation. He then received infliximab (5 mg/kg) concurrently with prednisone 50 mg which led to resolution of his symptoms after one infusion and successful rapid tapering of prednisone. Staging imaging after 2 months off pembrolizumab therapy (due to irEC) showed progression of his intracranial tumor and extracranial metastases. After an interruption of ICI for 4 months, it was decided to restart pembrolizumab with concurrent infliximab therapy. He subsequently received a total of twelve doses of pembrolizumab concurrently with infliximab (5mg/kg every 6 weeks, Table 2) over the course of 10.5 months. He did not experience any other irAEs or worsening rectal bleeding and a repeat flexible sigmoidoscopy showed mild active chronic colitis. Staging scans at that point showed stable intracranial and extracranial disease. Then, he developed *Clostridium difficile* colitis. He was treated with oral vancomycin to which he appropriately responded. However, after a few days of normal bowel movements, he started having loose bloody bowel movements and abdominal pain prompting an admission to the hospital. During that admission, he tested negative for *Clostridium difficile* and underwent a flexible sigmoidoscopy that showed severe colonic inflammation thought to be due to irEC. He received vancomycin, high dose intravenous steroids followed by oral steroids, and one infusion of infliximab (10 mg/kg) leading to symptom improvement. His steroids were tapered but therapy with pembrolizumab was discontinued. One month later, he developed retroperitoneal bleeding and was transitioned to hospice care.

**Table 1:** Patient characteristics, ICI treatment history, symptomatology, and endoscopy findings

Patient	Age	Sex	Malignancy	History of other ICI exposure	ICI type and dose	Days (doses) to onset of symptoms post ICI	Diarrhea grade	Other symptoms	Colitis grade	Endoscopic features	Histopathologic features
1	75	M	Meningioma	None	Pembrolizumab *Dose: 3 mg/kg* *Frequency:* every 3 weeks	39 days (2)	1	None	2	Colonoscopy: Sigmoid colon: localized moderate inflammation characterized by altered vascularity, congestion (edema), friability and granularity	Colonoscopy: - Ileum: mucosa with hyperplastic Peyer’s patches and no diagnostic abnormality - Ascending colon: mucosa with lymphoid aggregate and no diagnostic abnormality - Sigmoid colon: moderately active colitis with neutrophilic cryptitis and crypt abscesses
2	58	F	Colon	- Pembrolizumab (stopped 2 years prior to current ICI): no adverse effects but disease progression	Ipilimumab/Nivolumab *Dose:* Ipilimumab-1 mg/kg, Nivolumab- 240 mg (3 mg/kg) *Frequency:* combined every 6 weeks (4 doses total) followed by nivolumab alone every 2 weeks	8 days (1)	2	Abdominal pain	2	Upper endoscopy: - Gastric antrum: diffuse moderately erythematous mucosa without bleeding - Duodenum: an acquired benign-appearing, intrinsic moderate stenosis in the first portion of the duodenum	Upper endoscopy: - Gastric antrum/fundus/body: active chronic gastritis - Duodenum: mucosa with ulceration, crypt dropout, marked expansion of lamina propria with prominent eosinophils and acute inflammation - Duodenal stricture: mucosa with mild expansion of the lamina propria
3	70	F	Melanoma	- PD-L1 inhibitor (as a part of a clinical trial): for a total of 1 year (stopped 3 years prior to current ICI). No adverse events but disease recurrence - Pembrolizumab: 200 mg 3 (mg/kg) every 3 weeks for total of 8 doses (stopped 1 year prior to current ICI): no adverse events but disease progression	Ipilimumab *Dose:* 3 mg/kg *Frequency:* every 3 weeks	35 days (2)	2	Nausea, vomiting	2	Upper Endoscopy: - Stomach: normal - Duodenum: diffuse moderately scalloped mucosa Flexible Sigmoidoscopy: - Colon: examined portion was normal	Upper Endoscopy: - Duodenum: diffuse active duodenitis with villous blunting, expansion of the lamina propria with mixed inflammation, and reactive epithelial changes - Stomach: antral mucosa with edema and mild patchy inflammation Flexible Sigmoidoscopy: - Colon: normal
4	73	M	Melanoma	Atezolizumab (in combination with cobimetinib): total of 13 cycles (stopped 2 weeks prior to current ICI)	Ipilimumab/Nivolumab *Dose:* Ipilimumab-3 mg/kg, Nivolumab-1 mg/kg *Frequency:* combined every 3 weeks	11 days (1)	2	Nausea, vomiting, abdominal pain	2	Upper Endoscopy: - Stomach: non-bleeding erosive gastropathy - Duodenum: diffuse mildly congested mucosa without active bleeding Colonoscopy: - Sigmoid and descending colon: discontinuous areas of nonbleeding ulcerated mucosa with no stigmata of recent bleeding	Upper Endoscopy: - Stomach: active gastritis with small stromal granuloma in antrum. Active gastritis with stromal histiocytes in the body - Duodenum: active duodenitis with villous injury Colonoscopy: - Descending colon: focal active colitis with stromal histiocytes - Colon and sigmoid ulcers: severely active colitis with ulceration
5	79	F	SCC	None	Cemiplimab *Dose:* 350 mg *Frequency:* every 3 weeks	14 (1 dose)	1	Nausea, vomiting	2	Upper Endoscopy: - Stomach: Non-bleeding erosive gastropathy - Duodenum: normal Flexible Sigmoidoscopy: - Colon: Inflammation characterized by congestion (edema), erythema and granularity	Upper Endoscopy: - Stomach: reactive gastropathy and intestinal metaplasia - Duodenum: normal Flexible Sigmoidoscopy: - Colon: mucosa with mildly increased cellularity of the lamina propria and epithelial injury. Focal acute inflammation is also noted, but there is no increase in apoptosis.

**Table 2:** IrEC management and outcomes

Patient	ICI type/dose	Initial management	Number of steroid tapering attempts	Infliximab Dose-frequency	Doses of infliximab to clinical remission	Doses of ICI concurrently administered with infliximab	Follow up endoscopy on concurrent treatment (months)	Recurrence	# of months of follow-up on ICI/on concurrent therapy	Disease progression/Follow up
1	Pembrolizumb *Dose: 3 mg/kg* *Frequency:* every 3 weeks	Prednisone 40 mg > 60 mg PO daily> taper failure +azithromycin + metronidazole	2	5 mg/kg - every 2 weeks for first 2 doses then every 6 weeks	1	12	Flexible Sigmoidoscopy (4 months): Endoscopic: erythematous mucosa in sigmoid, normal colon for 40 cm Histologic: Mild active chronic colitis	Patient developed *Clostridium difficile* colitis then flare of irEC. *Treatment:* - Infliximab 10 mg/kg *-* Methylprednisolone 1 mg/kg BID then prednisone 75 mg PO BID followed by a taper - PO vancomycin - Immunotherapy was discontinued	14.5/10.5	- Staging scans after concurrent therapy (12 doses) showed stable disease - Developed retroperitoneal bleed and was transitioned to hospice care
2	Ipilimumab/Nivolumab *Dose:* Ipilimumab-1 mg/kg, Nivolumab- 240 mg (3 mg/kg) *Frequency:* combined every 6 weeks (4 doses total) followed by nivolumab alone every 2 weeks	Prednisone 60 mg PO daily>taper	1	5 mg/kg - every 2 weeks for first 2 doses then every 4 weeks	1	3 doses of (ipilimuab+Nivolumab) and 12 doses of nivolumab alone	Upper endoscopy (3 months): Endoscopic: - Gastric body: localized mild inflammation characterized by erythema and friability - Duodenum: an acquired benign-appearing, intrinsic moderate stenosis was found in the second portion of the duodenum associated with a small erosion Histologic: - Gastric body: lymphocytic involvement of gastric pits - Duodenum: no diagnostic abnormality - Duodenal stricture: ulceration and expansion of lamina propria by mononuclear cells	No	12/7.5	- Staging scans after concurrent therapy (15 doses) showed stable disease and patient continues concurrent therapy - Developed mucositis/stomatitis that is being managed conservatively
3	Ipilimumab *Dose:* 3 mg/kg *Frequency:* every 3 weeks	Methylprednisolone 1 mg/kg IV twice daily >taper failure	1	5 mg/kg - every 4 weeks	1	2	Not done	No	6.5/3.5	- Staging scans showed stable bulk of disease after concurrent therapy (2 doses) with ongoing slight progression in one metastatic lesion in the lung - Developed skin rash (ipilimumab cutaneous toxicity) that was managed successfully with topical steroids
4	Ipilimumab/Nivolumab *Dose:* Ipilimumab-3 mg/kg, Nivolumab-1 mg/kg *Frequency:* combined every 3 weeks	Prednisone 60 mg daily>taper failure	1	5 mg/kg - every 4 weeks	1	3	Upper endoscopy (1 month): Endoscopic/histologic - Stomach: normal/chronic inactive gastritis - Duodenum Normal/normal Colonoscopy (1 month): - Sigmoid/transverse colon ulcers: fragments of colonic mucosa with crypt architectural disarray and mildly increased cellularity of the lamina propria. Colonoscopy (3 months) - Colonic mucosa with scattered crypt epithelial apoptosis and minimal crypt architectural distortion	No	5/3	- Staging scans showed interval progression of his disease in the chest, abdomen and pelvis.
5	Cemiplimab *Dose:* 350 mg *Frequency:* every 3 weeks	Prednisone 60 mg daily>taper failure	1	5 mg/kg - once	1	2	Not done	No	4/2.5	- Staging scans demonstrated interval decrease in the disease burden in the chest and lymph nodes - Patient developed radiation/checkpoint pneumonitis and was treated with high dose oral steroids

### Patient 2

Patient 2 is a 58-year-old woman with stage 4 microsatellite instability (MSI) high colon cancer who was diagnosed at the age of 50. At diagnosis, she underwent a right total colectomy and was treated with 12 cycles of folinic acid, fluorouracil and oxaliplatin (FOLFOX). Two years later she was found to have metastatic disease in the ovaries and underwent bilateral salpingo-oophorectomy. This was followed by 18 cycles of folinic acid, fluorouracil, irinotecan hydrochloride, and avastin (FOLFIRI/Avastin) for metastatic foci in the abdomen. Subsequently she was treated with pembrolizumab for a total of 5 months without adverse events but then stopped due to disease progression. She underwent cytoreductive surgery (CS) with hyperthermic intraperitoneal chemotherapy (HIPEC). Afterwards, she was on two clinical trials using targeted therapies for advanced colon cancer but had no response to therapy. She then entered a clinical trial testing combination therapy with ipilimumab (CTLA-4 inhibitor), nivolumab (PD-1 inhibitor), and radiation therapy. Eight days after receiving the first dose, she developed epigastric pain, grade 2 diarrhea, abdominal distention, urgency, and stomatitis. She underwent an upper endoscopy that showed active chronic gastritis and a duodenal stricture with active duodenal inflammation (Table 1). Based on these findings, she was initially treated with prednisone 60 mg that was tapered successfully and her ICIs were held for a total of 7 weeks. Due to fear of recurrent duodenitis and the need for continued immunotherapy for disease control she was treated with infliximab (5 mg/kg every 4 weeks, Table 2) and continued to receive three further doses of ipilimumab/nivolumab then biweekly nivolumab. Over the past 7.5 months of concurrent therapy, she has had no further sustained diarrhea and only had occasional episodes of epigastric abdominal pain and nausea. She developed no other irAEs. A follow up upper endoscopy showed improvement of the inflammatory findings (Table 2). Staging imaging for colon cancer showed stable disease without further progression.

### Patient 3

Patient 3 is a 70-year-old woman who was first diagnosed with melanoma at the age of 40 and underwent a surgical resection at the time. At the age of 62, she was diagnosed with recurrent metastatic melanoma to the bone and lungs. Over the past 9 years, she has been treated with multiple surgical resections, adjuvant radiation, adjuvant interferon, and talimogene laherparepvec (TVEC). Next, she was enrolled in two clinical trials and one of them included an anti PD-L1 agent as a part of the treatment regimen but had no response or side effects to either trial. She received eight cycles of pembrolizumab without adverse events but with continued disease progression. Next, she was treated with ipilimumab. Two weeks after the second cycle of ipilimumab, the patient developed diarrhea, vomiting, abdominal pain, and poor oral intake. She was admitted to the hospital and underwent an upper endoscopy that demonstrated patchy gastritis and diffuse active duodenitis with villous blunting (Table 1). A flexible sigmoidoscopy demonstrated no abnormalities grossly and histologically. Based on these findings, she was treated with methylprednisolone 1 mg/kg twice daily to which her symptoms improved but on transitioning to high dose oral glucocorticoids her symptoms recurred and did not respond to another challenge of intravenous steroids. She was administered one dose of infliximab (5 mg/kg) and her steroids were tapered subsequently. Restaging imaging at that point revealed stable disease on ipilimumab. She had been off ICI for 8 weeks due to irEC and it was determined that she would benefit from continued immunotherapy with concurrent infliximab (5 mg/kg every 4 weeks, Table 2). The patient subsequently received 2 cycles of ipilimumab (to complete a total planned course of 4 cycles) with monthly infliximab infusions (total of 4 doses). She had no further diarrhea or abdominal pain through her treatment. While on concurrent therapy, she developed a pruritic skin rash that was biopsied and thought to be a cutaneous manifestation of ipilimumab toxicity. This was managed successfully with topical steroids. Restaging scans after completion of a total of 4 planned cycles of ipilimumab showed stability in the majority of her disease with slight progression in one metastatic lesion in the lung.

### Patient 4

Patient 4 is a 73-year-old man who was diagnosed with metastatic melanoma to the peritoneum and lungs at the age of 72. At diagnosis he was treated with 13 cycles of atezolizumab (anti-PD-L1) and cobimetinib (MEK inhibitor) as well as radiation therapy to the abdomen. He tolerated these therapies well with no adverse events however his disease continued to progress. Next, he was treated with ipilimumab and nivolumab (Table 1). Eleven days after receiving his first cycle, he developed worsening abdominal pain, nausea, vomiting and decreased oral intake. He underwent an upper endoscopy that demonstrated active gastritis and active duodenitis with villous injury. A flexible sigmoidoscopy showed mucosal ulceration with biopsies demonstrating severe active colitis with ulceration. He was then treated with prednisone 60 mg daily with difficulty tapering due to symptom recurrence. Further doses of ipilimumab and nivolumab were held for a total of 2.5 months. Restaging scans were obtained at the time and showed ongoing progression of disease burden in the abdomen. The severity of his irEC and the risk of colonic perforation prompted the decision to initiate concurrent treatment with infliximab. The patient received three more cycles of ipilimumab and nivolumab with infliximab (5 mg/kg every 4 weeks, Table 2). He had no further diarrhea, abdominal pain, nausea, vomiting, or other manifestation of irAEs. Follow-up upper endoscopy after two doses of concurrent therapy showed chronic inactive gastritis and a normal duodenal mucosa, and a flexible sigmoidoscopy showed a mucosal ulcer that demonstrated crypt architectural disarray but with improvement from previously seen active colitis. A follow up colonoscopy after completion of a total of three doses of concurrent therapy showed scattered crypt epithelial apoptosis and minimal crypt architectural distortion. Staging scans after completion of a total of 4 cycles of ipilimumab and nivolumab (3 of which were on concurrent therapy) demonstrated interval progression of his metastatic disease burden in the chest, abdomen, and pelvis. He is being considered for surgical debulking to reduce the metastatic disease burden in the abdomen.

### Patient 5

Patient 5 is a 79-year-old woman who was diagnosed with a cutaneous squamous cell carcinoma with metastasis to the lungs and lymph nodes. She underwent surgical resection of the primary lesion as well as radiation therapy to involved lymph nodes. Simultaneously, she was treated with cemiplimab (PD-1 inhibitor). Two weeks after receiving the first dose of cemiplimab, she developed significant nausea, vomiting, and diarrhea. She was admitted to the hospital and underwent an upper endoscopy which demonstrated reactive gastropathy and intestinal metaplasia in the gastric mucosa. A flexible sigmoidoscopy was performed and showed increased cellularity of the lamina propria and epithelial injury with focal acute inflammation (Table 1). She was treated with high dose oral glucocorticoids, received a dose of infliximab (5 mg/kg, Table 2), and cemiplimab therapy was held for 6 weeks. Her nausea, vomiting, and diarrhea resolved. Subsequently, she received a total of 2 cycles of cemiplimab without recurrence of her gastrointestinal symptoms. She developed worsening shortness of breath and chest imaging revealed evidence of pneumonitis. This was thought to be secondary to radiation and exacerbated by immunotherapy. She was treated with prednisone 60 mg that was tapered successfully; however, further cemiplimab doses were held. Restaging scans demonstrated interval decrease in the size of metastatic foci in the lymph nodes and the chest.

## Discussion

The biological rationale for combining anti-TNFα therapies with ICIs comes from recent insights into the role of TNFα in tumor immunology. TNFα produced in the setting of anti-PD-1 blockage leads to impaired CD8+ tumor infiltrating T lymphocyte responses [17]. Additionally, TNFα increases activation-induced cell death in T cells, limiting their viability in the tumor microenvironment [18, 20]. In a mouse model of melanoma, concurrent treatment with anti-PD-1 and anti-TNFα led to improved anti-tumor responses [17]. More recently, in a murine colon cancer model, concurrent treatment with anti-TNFα and combined anti-CTLA-4 and anti-PD-1 improved survival when compared with double checkpoint inhibition treatment alone [18]. When colitis was concomitantly induced in the tumor bearing mice through dextran sodium sulfate (DSS), the mice that received anti-TNFα and double checkpoint inhibition had better colitis amelioration and improved overall survival [18]. By blocking TNFα both studies showed an increase in CD8+ T cells numbers and viability in the tumor microenvironment and draining lymph nodes [17, 18]. These findings add to a growing body of literature that implicates innate inflammation in tumor promotion [21–23].

TNFα plays as essential role in the pathogenesis of irEC. Patients with irEC have up-regulated mucosal TNFα and a local activation of the TNFα gene signature [18]. Additionally, mucosal TNFα levels predict irEC steroid responsiveness with higher mucosal TNFα levels predicting lower steroid responsiveness [24]. Multiple groups have previously reported on treatment of severe irEC with TNFα blokecrs [8, 14, 15, 25]. However, after receiving anti-TNFα, patients’ ICI therapy was usually discontinued. In this case series, we report our institutional experience with patients who received immunotherapy and anti-TNFα concurrently.

Patients 2 and 4 were both treated with a combination of ipilimumab and nivolumab while Patients 1, 3, and 5 received monotherapy with pembrolizumab, ipilimumab, or cemiplimab, respectively. Patients 2, 3, and 4 all received a different ICI months to years prior to the regimen immediately associated with colitis. Given that the irAE to some ICI may manifest up to 2 years after therapy [26], the irEC they experienced may have a mixed component due to previous exposure to multiple ICIs. The onset of symptoms was sooner and the severity was worse in the patients who received a combination of ipilimumab and nivolumab compared to those receiving a single agent ICI, consistent with findings reported in the literature previously [6, 26].

Patient 1 had a history of segmental colitis associated with diverticulitis (SCAD) prior to initiation of ICI. Overlapping features of ICI and SCAD were seen on the colonic biopsy. The pathophysiology of SCAD is incompletely understood but the syndrome is thought to overlap with IBD [27]. Some retrospective studies reported increased risk of irEC in patients with baseline active inflammatory and autoimmune diseases, placing the patient at a higher risk of irEC [7, 28].

On presentation with symptoms, all patients were initiated on glucocorticoid therapy for irEC and achieved good control. Patients 1, 3, 4, and 5 were unable to maintain symptom control with glucocorticoid tapering. The decision to resume ICI after irEC carries a serious risk of relapse and is often done on an individual basis [29]. Some studies have shown that 50–60% of these patients have relapse of irEC [14, 29]. In one study, after irEC resolution, ICIs were restarted concomitantly with vedolizumab, an α4β7 integrin inhibitor that blocks T cell trafficking to the gut, only one out of eight patients had recurrence of irEC [14]. Although vedolizumab is a reasonable approach for treatment of glucocorticoid refractory irEC, inhibition of T cell trafficking into the gut may be risky in patients with gastrointestinal malignancies (e.g. patient 2) where antitumor T cells would also require access to the gastrointestinal mucosa. Similarly, vedolizumab could inhibit responses to gastrointestinal metastases, which are found in approximately 5% of patients with melanoma and are often not seen on surveillance imaging [30, 31]. We favor infliximab as initial biologic therapy for irEC for these reasons, as well as the potential antitumor benefit associated with TNFα blockade.

The decision to initiate concurrent ICI and anti-TNFα therapy in our cohort was driven by an inability to taper steroids and concern for irEC recurrence. Infliximab was administered at a dose of 5 mg/kg. The frequency of infliximab infusions varied depending on ICI regimen and irEC severity. In general, patients were loaded on infliximab using standard dosing at weeks 0, 2, and 6. Maintenance therapy frequency was selected based on the assumption that patients receiving ongoing immunotherapy in the setting of irEC would behave like patients with severe IBD, and that they may require more frequent infliximab administration than the standard 8 week interval. In addition, for patient convenience, infliximab was infused on the same schedule as the immunotherapy, with infusions occuring on the same day, though the drugs were not given simultaneously.

After initiation of concurrent anti-TNFα and ICI therapy, all patients continued to receive ICI without recurrence of symptoms. Follow up endoscopies in Patients 1, 2, and 4 showed resolution of acute inflammatory features. Staging scans in Patients 1, 2, 3, and 5 after concurrent therapy demonstrated overall disease stability. Patient 4 had progression of his disease despite targeted therapy, immunotherapy, and radiation. He is currently being considered for surgical management for symptom control. After receiving anti-TNFα and 12 further doses of ICI with no disease progression, Patient 1 developed a *Clostridium difficile* infection after which he had recurrence of irEC. After treatment with immunotherapies, gastrointestinal disruption (e.g.*,* due to infection) may cause alteration in the gut microbiota and the local immune compartment, resulting in a breach in tolerance leading to irEC [4, 7].

Our experience adds to the growing animal literature showing that concurrent anti-TNFα and ICI therapy is safe, does not negatively impact tumor control and is associated with a better side effect profile. Importantly, patients were able to continue on immunotherapy. We suggest that concurrent anti-TNFα be considered in patients who develop severe irECs early in their immunotherapy course where additional immunotherapy is likely to provide a benefit, and where other treatment alternatives are either unavailable or have a low likelihood of providing benefit. Prospective data will be necessary, however, to clearly define populations where anti-TNFα concurrent with immunotherapy is both safe, and leads to improved tumor outcomes.

An ongoing phase I clinical trial (NCT03293784) is evaluating the safety and tolerability of treating metastatic melanoma with ICIs combined with either infliximab or certolizumab, a similar anti-TNFα agent. Our experience helps provide evidence of the safety of combination treatment with anti-TNFα and ICI, which we propose should accelerate the initiation of a phase II clinical trial to examine the impact of TNFα blockade on both irAEs and antitumor immunity.

## Data Availability

Not applicable.
